# The Use of Telemedicine and Drones in Rural Clinics on Remote Japanese Islands

**DOI:** 10.7759/cureus.79078

**Published:** 2025-02-16

**Authors:** Yuuki Araki, Akihisa Nakamura, Miki Yamauchi, Marino Hirata, Kazuhiko Kotani

**Affiliations:** 1 Community and Family Medicine, Jichi Medical University, Shimotsuke, JPN

**Keywords:** island medicine, population size, remote medicine, rural medicine, rural practice

## Abstract

Background: Due to the limitations of resources and geographical isolation from the mainland, the use of telemedicine and drones may improve healthcare systems on remote islands. The aim of this study was to survey the status of telemedicine and drones in rural clinics on remote Japanese islands.

Methods: All rural public clinics (RPCs) on remote Japanese islands, managed in the rural plan of prefectural governments, were mailed a questionnaire on the use of telemedicine and drones in 2022. The survey data of RPCs on smaller remote islands (with a population of <1000 people) and larger remote islands (with a population of ≥1000) were compared.

Results: A total of 89 RPCs (40%) responded completely to the questionnaire. Twenty-one (24%) RPCs on smaller remote islands (41%) used telemedicine more than those on larger remote islands (7%) (P<0.01). Although no RPCs used drones for drug and specimen transfer, 33 (37%) wanted to use drones for drug transfer and 30 (34%) wanted to use them for specimen transfer. The desire to use drones for specimen transfer tended to be high in RPCs on smaller remote islands (43%) compared to that of larger remote islands (24%) (P=0.06).

Conclusions: The nationwide status of the use of telemedicine and drones was observed in RPCs on remote Japanese islands, which showed that RPCs on smaller remote islands used telemedicine and tended to want to use drones for specimen transfer compared with those on larger remote islands. This could be useful to develop healthcare and daily practice with telemedicine and drones, while considering the island’s population size, on remote Japanese islands.

## Introduction

Due to the limitations of resources and geographical isolation from the mainland, ensuring healthcare systems for people living on remote islands is a crucial issue in the world [1-3]. A lot of countries have remote inhabited islands, and these countries have various healthcare policies in order to support such people [4-7]. Japan has 306 remote islands, on which approximately half a million people live [8]. Of the islands, 224 clinics have been managed as rural public clinics (RPCs) under the rural plan of prefectural governments of Japan for providing healthcare and daily practice [9].

The introduction of recent technologies, such as telemedicine and drones, may improve the healthcare systems for people living on remote islands [10-13]. Telemedicine has the advantage of offering medical care over distance and in a timely manner, whereas some problems, such as technical and equipment-related problems, are pointed out [10,11]. Drones have the advantage of being able to supply medical products, including drugs and blood specimens, in a short time by air routes, even in poorly accessible areas, while difficulties with long distances and bad weather are pointed out [12,13]. Although telemedicine and drones are thus assumed to be helpful for people living on remote islands, the use of telemedicine and drones in RPCs remains unexplored. Therefore, the aim of this study was to survey the status of telemedicine and drones in RPCs on remote Japanese islands. Additionally, as the island’s population size can affect the healthcare system, this study investigated whether the use of telemedicine and drones differs depending on the island’s population size [14].

## Materials and methods

Study design and materials

This cross-sectional survey was conducted with the approval of the Research Ethics Committee of Jichi Medical University (No. 22-138). The Ministry of Health, Labour, and Welfare of Japan reported the Current Status of Medical Services in the Remote Area 2021, which showed the information on RPCs in Japan [15]. There were a total of 224 RPCs on remote islands, managed in the rural plan of prefectural governments of Japan, and all the RPCs on remote islands were mailed a questionnaire in December 2022 [9]. The questionnaire was developed by experts and physicians working on remote islands. In this questionnaire, medical professionals were asked about information on full-time physicians and nurses, the use of telemedicine (using a cell phone, tablet, or personal computer) and the use of or the desire to use drones (e.g., underuse, wanted to use, or did not want to use) for drugs and specimen transfer. The concrete questions were the following: 1) Are there full-time physicians at your clinic?; 2) Are there full-time nurses at your clinic?; 3) Do your clinics use telemedicine?; 4) Which types of telemedicine does your clinic provide if available?; 5) If your clinic uses drones for drug transfer, do you want to use them?; and 6) If your clinic uses drones for specimen transfer, do you want to use them?

Information on the population of remote islands, where the RPC was located, was also used based on the 2020 Population Census of Japan, as the island’s population size can affect healthcare systems [14,16]. In this study, remote islands with a population of ≥1000 people were treated as large remote islands, while those with a population of <1000 people were treated as small remote islands [14]. The straight-line distances between the RPC on remote islands and the nearest hospital from the RPC were calculated using the ArcGIS Pro 3.0 software program (ESRI, Redlands, CA, USA).

In this study, three types were included as telemedicine: first, “Physician to Patient” is a type where a physician sees a patient through an electronic device for telemedicine, such as a smartphone, tablet, or personal computer. Second, “Physician to Patient with Nurse” indicates that while a nurse assists with the operation of devices near a patient, a physician sees the patient using telemedicine. Third, “Physician to Patient with Specialist” indicates that a physician and a patient consult with a specialist located on the mainland using telemedicine.

Statistical analysis

The data are reported as percentages for categorical variables and medians with interquartile ranges (IQR) for continuous variables. The characteristics of the RPCs on small remote islands and those on large remote islands were compared using the chi-square test for categorical variables and the Mann-Whitney U test for continuous variables. The SPSS software program (Version 27; IBM, Armonk, NY, USA) was used for statistical analyses. Statistical significance was set at P<0.05.

## Results

A total of 89 RPCs (40%) that completely responded to the survey were analyzed, and their characteristics of RPC are shown in Table 1. Regarding medical professionals, 58 RPCs (65%) had full-time physicians and 72 RPCs (81%) had full-time nurses. The median population of remote islands where the RPCs were located was 1054 (IQR: 196-2509). The median straight-line distances between RPCs and the nearest hospitals were 23 km (IQR: 14-49). Twenty-one RPCs (24%) used telemedicine. Of them, 19 RPCs indicated the type of telemedicine: "Physician to Patient with Nurse" was used by 14 (74%), "Physician to Patient" by four (21%), and "Physician to Patient with Specialist" by one (5%). No RPCs used drones for drug and specimen transfer. Thirty-three (37%) RPCs wanted to use drones for drug transfer in the future. Thirty (34%) RPCs in remote islands responded that they wanted to use drones or specimen transfer.

**Table 1:** Characteristics of rural public clinics on remote Japanese islands IQR: interquartile range

Characteristics	N=89
Clinics with full-time physicians, n (%)	58 (65%)
Clinics with full-time nurses, n (%)	72 (81%)
Population of remote islands with clinics, n (IQR)	1054 (196–2509)
Distance between a clinic and the nearest hospital, km (IQR)	23 (14–49)
Telemedicine use, n (%)	21 (24%)
Drone use	0 (0%)
Desire to use drones for drug transfer, n (%)	33 (37%)
Desire to use drones for specimen transfer, n (%)	30 (34%)

**Figure 1:**
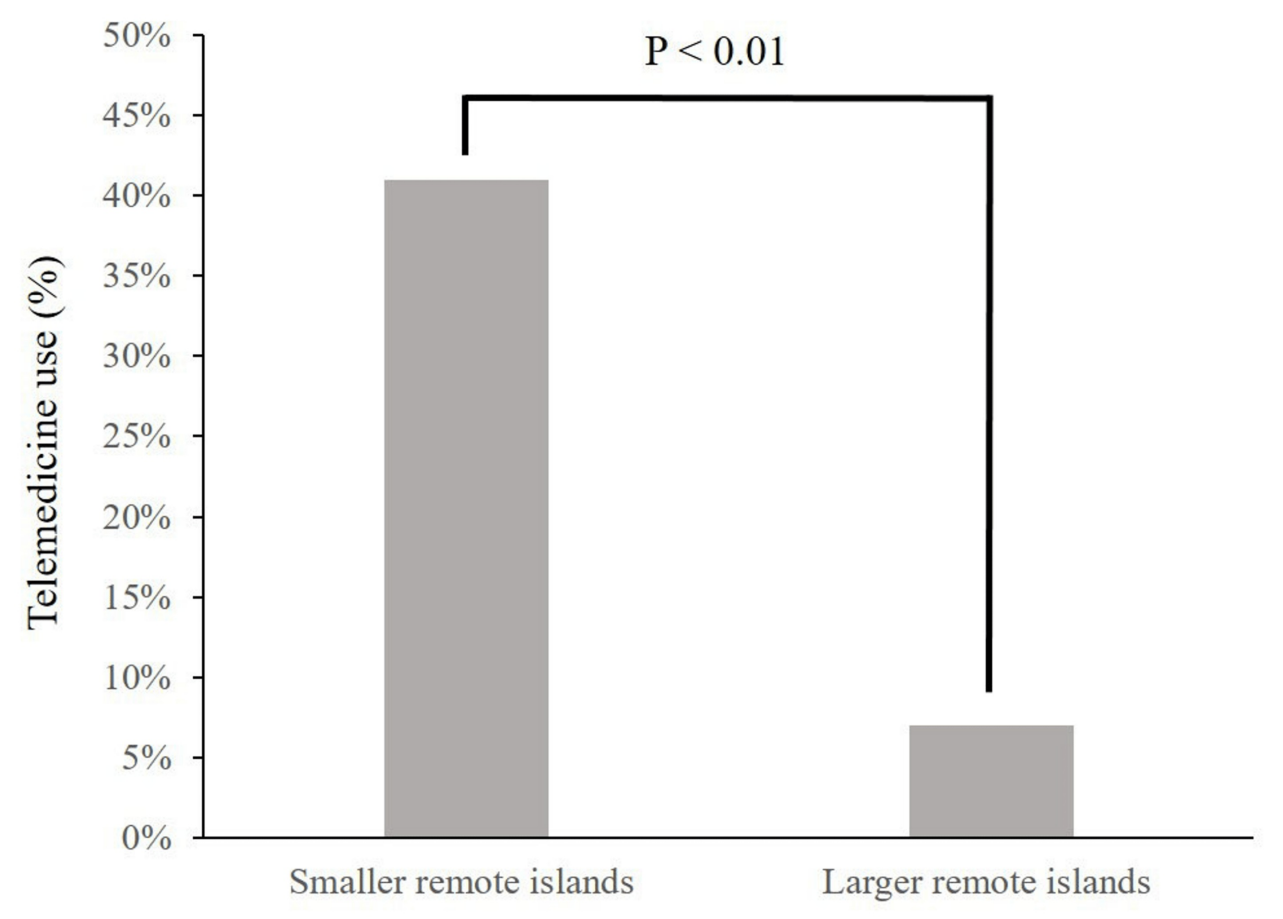
Comparison of the proportion of RPCs using telemedicine between smaller remote islands and larger remote islands RPC: rural public clinic

**Figure 2:**
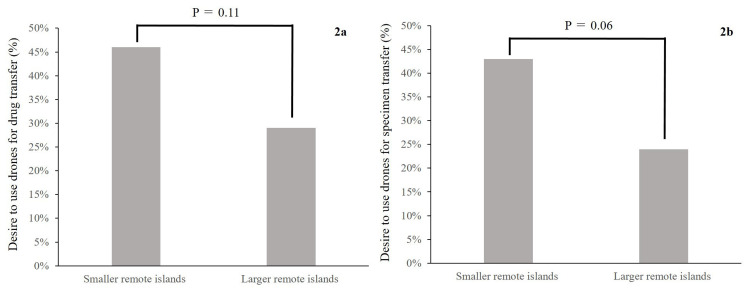
Comparison of the proportion of RPCs wanting to use drones between smaller remote islands and larger remote islands. Drones for drug transfer in 2a and specimen transfer in 2b RPC: rural public clinic

As shown in Table 2, there was no difference in the proportion of full-time physicians between RPCs on smaller and larger remote islands. While the proportion of full-time nurses was deemed to be high in RPCs on smaller remote islands, there was no difference in their proportion between RPCs on smaller and larger remote islands. The RPCs on smaller remote islands (41%) used telemedicine more than those on large remote islands (7%) (P<0.01) (Figure 1). The proportion of RPCs that wanted to use drones for specimen transfer tended to be higher on smaller remote islands (43%) compared with that of large remote islands (24%) (P=0.06) (Figure 2).

**Table 2:** Comparison of smaller remote islands and larger remote islands in RPCs on Japanese remote islands ^a ^Chi-square test. ^b^ Mann–Whitney U test. ^*1^ p< 0.05. IQR: interquartile range; RPC: rural public clinic

	Smaller remote islands (n=44)	Larger remote islands (n=45)	P-value
Full-time physicians, n (%)	29 (66%)	29 (64%)	0.89
Full-time nurses, n (%)	39 (89%)	33 (73%)	0.07
Population, n (IQR)	194 (117–277)	2509 (1935–16119)	＜0.01 ^b,*1^
Distance, km (IQR)	23.0 (15.8–43.8)	19.0 (11.4–56.3)	0.58
Telemedicine use, n (%)	18 (41%)	3 (7%)	＜0.01 ^a,*1^
Desire to use drones for drug transfer, n (%)	20 (46%)	13 (29%)	0.11
Desire to use drones for specimen transfer, n (%)	19 (43%)	11 (24%)	0.06 ^a^

## Discussion

In this study of the current status of telemedicine and drones in RPCs on remote Japanese islands, approximately one-quarter of the RPCs were found to use telemedicine. More RPCs used telemedicine on smaller remote islands than on larger remote islands. While no RPCs used drones, approximately one-third of the RPCs wanted to use drones in the future. There was a tendency for RPCs that wanted to use drones for specimen transfer, which was high on smaller remote islands compared with larger remote islands. This could be a useful perspective to provide healthcare and daily practice with telemedicine and drones on remote Japanese islands.

The use of telemedicine in one-quarter of the RPCs on remote islands was noted. A previous report as of 2020 demonstrated that 19% of rural clinics in mainland and remote islands used telemedicine as consultations for patients [17]. Although the previous report is not directly compared with our result of remote islands, the greater use of telemedicine may partly imply a greater need for telemedicine in RPCs on remote islands [16]. In addition, “Physician to Patient with Nurse” accounted for the most common type of telemedicine used in RPCs on remote islands. This suggests that this type is suitable for such PRCs because there is an increase in the aging of the population on remote islands and many older adults are not very competent with the use of devices necessary to conduct telemedicine requiring the support of nurses [18,19].

Of note is that telemedicine was used more on smaller remote islands than on larger remote islands. Some speculations have been raised regarding this result. In general, telemedicine, which is connected to resources in the judgment of clinical practice and consultation, is helpful in cases of specialized care and transferring the patient and/or physician to other regions and/or the mainland [20-22]. The cases might exist on smaller remote islands, which seem to have limited resources such as medical staff and specialized care facilities. Furthermore, telemedicine may be used as a tool to deal with situations where physicians temporarily leave remote islands. On larger remote islands, there are other medical institutions in addition to RPCs; in this case, even if the physician at the RPC is temporarily absent, patients may be able to visit other medical institutions. Emergent situations are especially reported to be helpful for telemedicine [23,24]. The detailed reasons for more use of telemedicine on smaller islands must therefore be further investigated.

Although no RPCs used drones, approximately one-third of RPCs on remote islands wanted to use drones for drug and specimen transfer in the future. This would mean an expectation in association with future drone applications. It might be partly due to the fact that the drugs and devices necessary for testing may be lacking on remote islands, as the use of drones enables us to correspond to sudden drug shortages and transport specimens that need to be tested rapidly [12,13]. Especially, RPCs on smaller remote islands tended to have a positive attitude regarding their use for specimen transfer, suggesting that a greater need for drones exists on smaller remote islands as described in a report that devices for testing were limited on smaller islands [25]. Anyway, future drone application is a point that should be deepened on remote islands.

The strength of this study is that this questionnaire survey was conducted nationwide at RPCs on remote Japanese islands, which could be representative data for a country. Nevertheless, this study is associated with some limitations. First, the response rate of the questionnaire was not high. The response rate by mail survey is not always high, which is an issue to increase the rate [17]. The information on non-responders to the questionnaire may also be worthwhile. Second, the survey was self-reported. Third, we did not investigate the age of physicians and the climate, which may be considered as confounding factors of this study [26,27]. Especially, younger physicians are reported to be more likely to favor recent technologies, such as telemedicine and drones [26]. The frequency of bad weather affects the visit of hospitals and transport to the mainland [27]. Fourth, we did not investigate the diseases used for telemedicine. These limitations should be taken into careful consideration when generalizing the results. Research on the actual use of telemedicine and its effect on clinical practice, as well as the concrete items of tests wanted for drones will be the next plan. As the technical, legal, and economic issues are still unsolved for the promotion of telemedicine and drones, such challenges (i.e., their easy and inexpensive use) will also be included in future research [17,28,29].

## Conclusions

In this study, the current status of telemedicine and drones at RPCs on remote Japanese islands was observed using a questionnaire survey. Approximately one-quarter of the RPCs used telemedicine, and more RPCs used telemedicine on smaller remote islands compared to larger remote islands. While no RPCs used drones for drug and specimen transfer, approximately one-third of the RPCs wanted to use drones in the future. The desire to use drones for specimen transfer tended to be high in RPCs on smaller remote islands compared to those on larger remote islands. This gives insight into the provision of healthcare and daily practice with telemedicine and drones, while considering the island’s population size, among remote Japanese islands.
